# Assessment of Epigenetic and Phenotypic Variation in *Populus nigra* Regenerated via Sequential Regeneration

**DOI:** 10.3389/fpls.2021.632088

**Published:** 2021-07-06

**Authors:** Weixi Zhang, Yanbo Wang, Shu Diao, Shanchen Zhong, Shu Wu, Li Wang, Xiaohua Su, Bingyu Zhang

**Affiliations:** ^1^State Key Laboratory of Tree Genetics and Breeding, Research Institute of Forestry, Chinese Academy of Forestry, Beijing, China; ^2^Key Laboratory of Tree Breeding and Cultivation of State Forestry and Grassland Administration, Research Institute of Forestry, Chinese Academy of Forestry, Beijing, China; ^3^Nanchang Institute of Technology, Nanchang, China

**Keywords:** sequential regenerants, MSAP, DNA methylation, epigenetic variation, *Populus nigra*

## Abstract

Somatic variation has been demonstrated in tissue culture regenerated plants of many species. In the genus *Populus*, phenotypic variation caused by changes in 5-methylcytosine within the plant genome have been reported. To date, the phenotypic and epigenetic stability of plants regenerated from sequential regeneration has not been tested in trees. In this study, we detected DNA methylation of CCGG sites in regenerated plants of five generations in *Populus nigra* using methylation-sensitive amplified polymorphisms, and evaluated their growth performance and physiological traits. About 10.86–26.80% of CCGG sites in the regenerated plant genome were demethylated and 5.50–8.45% were methylated, resulting in significantly lower DNA methylation levels among all regenerated plants than among donor plants. We detected a significant difference in methylation levels between first regeneration regenerated plants (G1) and those of the other four generations (G2–G5); there were no significant differences among the four later generations. Therefore, the dramatic decrease in DNA methylation levels occurred only in the first and second poplar regenerations; levels then stabilized later in the regeneration process, indicating that two regeneration events were sufficient to change the methylation statuses of almost all CCGG sites sensitive to regeneration. Differences in growth and physiological traits were observed between regenerated plants and donor plants, but were significant only among plants of certain generations. Significant correlations were detected between methylation level and transpiration rate, net photosynthetic rate, peroxidase activity, and instant water utilization efficiency, indicating the involvement of epigenetic regulation in this unpredictable phenotypic variation.

## Introduction

*In vitro* plant cell and tissue culture techniques are the basis of micropropagation and genetic transformation programs (Miguel and Marum, 2011; Rathore et al., 2012). Phenotypes of plants clonally propagated through tissue culture sometimes differ from those of the mother plant, especially in plants regenerated from callus in plant species such as rye (de la Puente et al., 2008), potato (Bordallo et al., 2004), pea (Kuznetsova et al., 2006), cocoa (Rodríguez López et al., 2010), and poplars (Saieed et al., 1994; Rani et al., 1995). Such somaclonal variation is thought to be caused by *in vitro*-induced stress and the breakdown of normal cellular processes (Phillips et al., 1994; Miguel and Marum, 2011). At the molecular level, somaclonal variation has been shown to be caused by DNA mutation and epigenetic changes, specifically by changes in genomic DNA methylation (Kaeppler et al., 2000; Li et al., 2007; Gao et al., 2010; Rodríguez López et al., 2010). Methylation instability produced via *in vitro* plant regeneration has been reported in many plant species, including maize (Kaeppler and Phillips, 1993; Stelpflug et al., 2014), potato (Dann and Wilson, 2011), barley (Li et al., 2007; Orłowska et al., 2016), cocoa (Rodríguez López et al., 2010), oil palm (Kubis et al., 2003), and poplars (Vining et al., 2013). The transfer of DNA methylation changes from regenerated plants to their progenies has been observed in maize (Stelpflug et al., 2014) and rice (Stroud et al., 2013), indicating that some epigenetic changes are stable and heritable. Therefore, somaclonal variation could be used to select crops with traits that are more advantageous than those of the mother plant. In contrast, somaclonal variation can be an obstacle to maintaining genetic fidelity, as required in clonal propagation or transgenic research.

In the past two decades, studies have shown that the frequency of somaclonal variation can change over time and with repeated subculturing. For example, leaves of late-regenerated plant cocoa exhibited significantly less genetic and epigenetic divergence from source leaves than those exposed to short periods of callus growth (Rodríguez López et al., 2010); hops derived from third-callus subcultures showed higher genetic distance from *in vitro* control plants than those from the first and second subcultures (Peredo et al., 2009). Recently, by Agrobacterium-mediated genetic transformation of hypocotyls and negative plant regeneration at first step, and then negative plant underwent 4 times of sequential plant regeneration, an elite cotton Jin668, with an extremely high regeneration ability was developed from an inbred cultivar Y668; and the average cytosine methylation levels of protein-coding genes, transposon elements and TE-related genes in CG, CHG and CHH contexts in leaves of Jin668 were different from that of the Y668 (Li et al., 2019).

Although phenotypic variation and cytosine DNA methylation variation in regenerated plants have been reported in poplars (Saieed et al., 1994; Vining et al., 2013), to our knowledge, DNA methylation changes and their corresponding phenotypic variations have not been reported in trees for during multiple sequential regenerations. The objectives of this study were to (i) use the MSAP method to detect 5-methylcytosine changes in *Populus nigra* plants produced via five regenerations, (ii) assess the occurrence and extent of phenotypic instability induced by repeated plant regeneration.

## Materials and Methods

### Plant Materials

**FIGURE 1:**
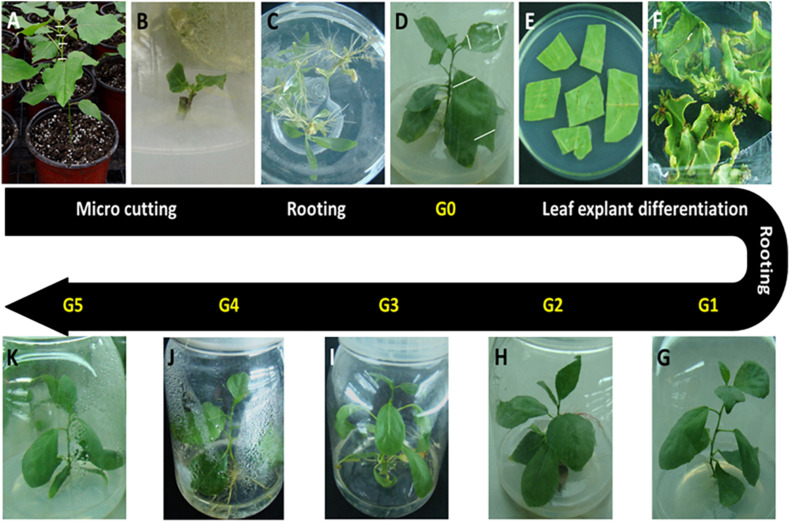
Plant materials used in this study. All *in vitro* materials were derived from the *Populus nigra* clone “N46.” **(A)** Starting material. Stem cuttings (hash marks) were cut from plants grown in a greenhouse. **(B)** Stem cuttings were cultured on hormone-free Murashige and Skoog medium for 2–3 weeks until axillary buds matured. **(C)** Shoots were cultured on rooting medium for 4 weeks. **(D)** Plants were grown to 7–8 cm and designated as donor plants (G0). **(E)** Leaf discs were cut from G0 plants and cultured on shoot induction medium. **(F)** Adventitious shoots initiated| from calli formed on leaf cutting edges. **(G)** Adventitious shoots were grown to 0.5–0.8 cm, cut, and cultured on rooting medium to produce G1 plants. G1 leaf explants were cultured as described above to obtain G2 **(H)**, G3 **(I)**, G4 **(J)**, and G5 **(K)** plants by repeating this process.

The *P. nigra* clone “N46” was propagated from stem cuttings and used for *in vitro* plant production. New semi-ligneous stem nodes of clone “N46” were sterilized with 10% sodium hypochlorite for 5 min and cultured on hormone-free Murashige and Skoog (MS) medium at 28°C and a 16-h photoperiod. We cut and cultured 2-cm axillary buds on rooting medium (1/2 MS + 0.01 mg/L indole-3-butyric acid + 0.01 mg/L naphthaleneacetic acid [NAA]). These plants were designated as donor plants (G0) (Figures 1A–D). We then cut leaf explants (about 1 cm^2^) from G0 plants and cultured these first in shoot induction medium (MS + 0.5 mg/L 6-benzylaminopurine + 0.03 mg/L NAA) in the dark at 28°C for 1 day, and then under a 16-h photoperiod at the same temperature (Figure 1E). Regenerated shoots (0.5–0.8 cm) were cut and transferred to rooting medium; plants thus obtained were designated as G1 plants (Figures 1F,G). G1 leaf explants were cultured as described above, and G2–G5 plants were ultimately obtained by repeating this process (Figure 1G–K).

Four plants were randomly selected from each generation (G0–G5) and three mature leaves (the 4th to 6th leaves form the top) were collected from each plant. These leaves were frozen immediately in liquid nitrogen, stored at −80°C, and used for DNA extraction.

### DNA Extraction and MSAP Analysis

Total genomic DNA was isolated from 100 mg of leaves of four biological replicates of each generation using DNeasy plant mini kit (QIAGEN, Hilden, Germany) according to the manufacturer’s instructions. The integrity, purity, and quality of total DNA were determined using 1.2% agarose gel electrophoresis and a NanoDrop 8000 spectrophotometer (Thermo Scientific, Waltham, MA, United States). Only qualified DNA samples (1.8 < A260/280 < 2.0, 2.0 < A260/230 < 2.3) were used for further analysis.

The MSAP analysis was performed as described by Diao et al. (2016). Briefly, 450ng genomic DNA of each sample was digested with restriction enzyme combinations *Eco*RI (10U)/*Hpa*II (5U) and *Eco*RI (10U)/*Msp*I (5U) (New England Biolabs, United States), respectively at 37°C for 12 h and deactivated at 65°C for 20 min. All digested DNA were ligated to the adapters using T4 DNA ligase (New England Biolabs, United States) at 16°C for 16 h. The reaction was stopped by incubating the mixture at 65°C for 20 min. The ligated DNA product above was used for pre-selective amplification reaction in a Applied Biosystems^®^ Veriti^®^ Thermal Cycler (ABI, United States). After checking for the smear of fragments (100–1,000 bp in lengths) in 2% agarose gel electrophoresis, the qualified pre-amplification mixture was diluted to 20-fold in deionized water and used as templates for the selective amplification. A touchdown PCR program was used in selective amplification reaction in Applied Biosystems^®^ Veriti^®^ Thermal Cycler (ABI, United States). The digestion, ligation and amplification reactions in all the DNA samples were carried out simultaneously to reduce the experimental error.

The selective amplification DNA products were separated by capillary electrophoresis on GeXP Genetic Analysis System (Beckman, American). Fragment analysis module of GeXP software was used to analysis the raw data, fragments within 50–700 bp range were marked, exported with 1 (presence of bands) or 0 (absence of bands) for further data analysis.

The amplified DNA fragments were divided into four types: Type I, product amplified by both enzymes combinations. i.e., *Eco*RI/*Hpa*II and *Eco*RI*/Msp*I, indicating the non-methylated site. Type II, band appeared only in *Eco*RI*/Hpa*II but not in the *Eco*RI/*Msp*I, indicating the hemimethylated state of DNA that resulted from methylation in one DNA strand but not in its complementary strand. Type III, band generated in *Eco*RI*/Msp*I but not in the *Eco*RI*/Hpa*II, represents the case of full CG (internal cytosine) methylation. Type IV, no products amplified by any enzyme combination, represents the case of full methylation at both cytosines. Percentage of methylation was calculated using the following formula (Karan et al., 2012):

Totalmethylationlevel(%)=[(II+III+IV)/(I+II+III+IV)]× 100%;

Fullymethylationlevel(%)=[(III+IV)/(I+II+III+IV)]×100%;

Hemi-methylationlevel(%)=[(II)/(I+II+III+IV)]×100%.

### Growth and Physiological Parameter Measurements

We planted six clones (four that were also used for MSAP analysis and two randomly selected from among unused regenerated plants) from each generation in a greenhouse in Beijing, China (46°44°N, 117°10°W) in March. Greenhouse conditions included controlled temperature (24–30°C) and natural sunlight; three replicates were performed. Plants were rotated every 2 weeks. At the beginning of August, we measured peroxidase (POD) and superoxide dismutase (SOD) activity, chlorophyll content, and photosynthetic capacity of mature leaves (third or fourth mature leaves). The height and basal diameter of these plants were measured at the end of October.

We performed a POD activity assay following the method of Cakmak and Marschner (1992) with minor modifications. Fresh leaf samples (200 mg) were homogenized in 3 mL of ice-cold potassium phosphate buffer (50 mM, pH 6.8) using a pre-chilled mortar and pestle, and the supernatant was obtained by centrifugation at 12,000 rpm at 4°C for 20 min. The reaction mixture was prepared by mixing 0.5 mL supernatant, 2.5 mL potassium phosphate buffer (50 mM, pH 6.8), 1 mL guaiacol (50 mM), and 1 mL H_2_O_2_ (0.2%). The optical density was recorded at 470 nm using a microplate reader (SpectraMax i3x, Molecular Devices, Austria). One unit of POD activity was defined as an increase of 0.01 A_470_/min.

We performed a SOD activity assay following the method of Baby and Jini (2011) with minor modifications. Fresh leaf extraction was performed as described above. The reaction mixture was prepared using 0.3 mL methionine (130 mM), 0.3 mL nitro-blue tetrazolium (0.75 mM), 0.3 mL EDTA (0.1 mM), 1.5 mL potassium phosphate buffer (50 mM, pH 7.8), 0.3 mL riboflavin (0.02 mM), and 0.1 mL of fresh sample extract. The mixture was then incubated under 4,000-lux light at 25°C for 40 min. Absorbance was recorded at 560 nm using a microplate reader (SpectraMax i3x, Molecular Devices, Austria). One unit of enzyme was defined as the amount of enzyme corresponding to 50% SOD inhibition.

Chlorophyll content was determined according to a modified method outlined by Arnon (1949). Leaf samples (0.2 g) were soaked in 10 mL of 80% acetone solution for 16 h in the dark, shaken, and mixed several times until all leaves turned white. Solution absorbance was recorded at 663 and 645 nm using a microplate reader (SpectraMax i3x, Molecular Devices, Austria). Total chlorophyll content was then calculated as follows:

$$\text{C}\,\left(\text{mg}/\text{g}\right)=\left[20.2\,\left(A_{645}\right)+8.02\,\left(A_{663}\right)\right]\times \left(V/1000\,W\right).$$

where C is total chlorophyll content (mg/g); *A*_645_ and *A*_663_ are solution absorbance at 645 and 663 nm, respectively, *V* is the total extract volume; and *W* is leaf fresh weight.

Photosynthetic rate (Pn), transpiration rate (Tr), stomatal conductance (Gs), intercellular CO_2_ concentration (Ci), and instant water use efficiency (WUE) were measured using a portable infrared gas analyzer (LI-6400; LI-COR, Lincoln, NE, United States) at a temperature of 25°C and light intensity of 1,500 μmol/m^2^s.

All statistical analyses were performed using the SPSS v. 17.0 software (SPSS Inc., Chicago, IL, United States). Differences were evaluated using two-tailed analysis of variance with Duncan’s multiple range test (*P* < 0.05). Relationships between physiological parameters and methylation levels were evaluated using correlation analysis based on Pearson’s correlation coefficient.

## Results

### DNA Methylation Levels Decreased Dramatically in *P. nigra* Regenerated Plants During Multiple Regenerations

We used 22 primer combination pairs to determine cytosine methylation statuses at CCGG sites of mature leaves from G0–G5 plants. A total of 938–940 DNA fragments were amplified from DNA extracted from leaves from each generation (Table 1). Each primer combination produced 20–75 bands (average: 47), with the primer combination E3-HM17 producing the most bands (*n* = 75), and E1-HM27 and E2-HM24 producing the fewest (*n* = 20) (Supplementary Figure 1). Band lengths ranged from 50 to 700 bp.

**TABLE 1:** Change in *P. nigra* N46 DNA methylation levels among regenerated plants of five generations compared with those of donor plants.

**MSAP band type**	**G0**	**G1**	**G2**	**G3**	**G4**	**G5**
I	442 ± 19	488 ± 11	613 ± 15	616 ± 7	646 ± 6	631 ± 13
II	194 ± 16	151 ± 4	98 ± 8	86 ± 8	66 ± 8	85 ± 7
III	213 ± 3	204 ± 2	153 ± 4	166 ± 10	155 ± 9	167 ± 10
IV	90 ± 3	95 ± 8	76 ± 6	71 ± 7	72 ± 3	56 ± 8
Total	939	938	940	939	939	939
T (%)^a^	52.96 ± 2.05a	48.03 ± 1.26b	34.75 ± 1.60c	34.43 ± 0.71c	31.20 ± 0.65c	32.84 ± 1.37c
F (%)^b^	32.27 ± 0.28a	31.91 ± 0.81a	24.35 ± 1.07b	25.24 ± 0.54b	24.14 ± 1.23b	23.75 ± 1.89b
H (%)^c^	20.70 ± 1.81a	16.12 ± 0.46b	10.40 ± 0.87c	9.19 ± 0.85c	7.06 ± 0.87c	9.09 ± 0.73c

*^*a*^Total methylation level (%) = [(II + III + IV)/(I + II + III + IV)] × 100%. ^*b*^Fully methylation level (%) = [(III + IV)/(I + II + III + IV)] × 100%. ^*c*^Hemi-methylation level (%) = [(II)/(I + II + III + IV)] × 100%. Type I indicated absence of methylation due to the presence of bands in both EcoRI/HpaII and EcoRI/MspI digest; type II bands appeared only in EcoRI/HpaII digestion but not in the EcoRI/MspI digest; type III generated bands obtained in EcoRI/MspI digest but not in the EcoRI/HpaII digest; and type IV represents the absence of band in both enzyme combinations. The different letters after data means significant deferences at *P* < 0.05.*

We calculated the DNA methylation levels of the G0–G5 plants; differences in methylation levels were observed not only between donor plants (G0 plants) and regenerated plants, but also among the five generations. The highest total DNA methylation level was observed in G0 plants, followed by G1; a sharp decrease was observed in G2, followed by relative stability in G3, G4, and G5 plants. A similar trend was observed at total and hemi-methylation levels (Table 1), which were significantly lower in all regenerated plants than in the donor plants. Except for G1 plants, total methylation levels of all regenerated plants were lower than those of donor plants. Significant differences in methylation level were also observed between regenerated plants in all five generations, with those of G1 plants significantly higher than those of plants in the other four generations; no significant differences were observed among plants of the later four generations. Thus, DNA methylation levels of poplar regenerated plants became stable only after two generations; the plant regeneration process significantly decreased genomic DNA methylation levels in poplar regenerated plants, especially those of the first and second generation, after which this effect became weaker.

### Regeneration Processes Induced Both Methylation and Demethylation in *P. nigra* Regenerated Plants of Five Subsequent Generations

Based on MSAP data, all possible banding patterns among regenerated plants of the five generations were compared to those of donor plants to identify changes in cytosine methylation patterns during the process of five sequential regenerations. A total of 16 banding patterns were observed (Table 2). Patterns A–D represent monomorphic classes, in which methylation patterns were the same in G0 plants and regenerated plants in five subsequent generations. Patterns E–J indicate cytosine demethylation, and patterns K–P indicate possible cytosine methylation events induced by regeneration processes (Karan et al., 2012).

**TABLE 2:** Analysis of DNA methylation patterns in regenerated plants of five generations compared with donor plants.

**Description of patten**	**Class**	**Banding patten**				**G1**	**G2**	**G3**	**G4**	**G5**
		**G0**		**G1-G5**						
		***Hpa*II**	***Msp*I**	***Hpa*II**	***Msp*I**					
No Change	A	1	0	1	0	122 ± 12	58 ± 1	56 ± 1	47 ± 1	57 ± 8
	B	0	1	0	1	178 ± 2	129 ± 3	143 ± 4	129 ± 2	130 ± 5
	C	1	1	1	1	400 ± 12	419 ± 20	412 ± 14	425 ± 18	406 ± 19
	D	0	0	0	0	58 ± 5	48 ± 4	44 ± 3	44 ± 3	37 ± 3
		Total percentage (%)				758 ± 5 80.69 ± 0.57	655 ± 18 69.76 ± 1.87	656 ± 15 69.83 ± 1.61	645 ± 16 68.69 ± 1.67	630 ± 15 67.09 ± 1.57
Demethylation	E	1	0	1	1	59 ± 2	126 ± 13	127 ± 15	133 ± 18	132 ± 14
	F	0	1	1	1	24 ± 5	64 ± 9	70 ± 1	81 ± 6	84 ± 5
	G	0	0	1	1	0	0	0	0	0
	H	0	1	1	0	1 ± 1	15 ± 9	4 ± 2	3 ± 1	6 ± 2
	I	0	0	1	0	0	0	0	0	0
	J	0	0	0	1	18 ± 2	25 ± 2	24 ± 4	25 ± 3	30 ± 2
		Total percentage (%)				102 ± 5 10.86 ± 0.56	230 ± 15 24.49 ± 1.60	224 ± 17 23.89 ± 1.82	242 ± 15 25.81 ± 1.58	252 ± 19 26.80 ± 2.00
Methylation	K	1	1	1	0	25 ± 1	15 ± 2	19 ± 5	9 ± 1	17 ± 3
	L	1	1	0	1	15 ± 3	7 ± 3	9 ± 2	8 ± 2	16 ± 9
	M	1	1	0	0	3 ± 2	2	3 ± 2	1 ± 1	4 ± 2
	N	1	0	0	1	2 ± 1	4 ± 1	5 ± 2	7 ± 3	6 ± 1
	O	1	0	0	0	16 ± 5	9 ± 3	13 ± 3	13 ± 2	8 ± 3
	P	0	1	0	0	18 ± 4	17 ± 3	10 ± 3	14 ± 1	7 ± 1
		Total percentage (%)				79 ± 4 8.45 ± 0.40	54 ± 6 5.75 ± 0.59	59 ± 4 6.28 ± 0.44	52 ± 5 5.50 ± 0.58	57 ± 13 6.11 ± 1.38

We detected similar proportions of CCGG sites in regenerated plants among the five generations and in donor plants (67.09–80.69%; Table 2). More demethylated sites were detected in plants of later generations (G2–G5) than in G1, whereas more methylated sites were detected in G1 plants than in later generations. The percentage of sites demethylated during the regeneration process was much higher than the percentage of methylated sites among all regenerated plants among the five generations, resulting in an overall demethylation trend among *P. nigra* regenerated plants. These results indicate that DNA demethylation and methylation events occurred during the poplar regeneration process, with demethylation generally dominating.

### Phenotypic Variation Among Generations and Correlation Between Phenotype and Methylation Level

**FIGURE 2:**
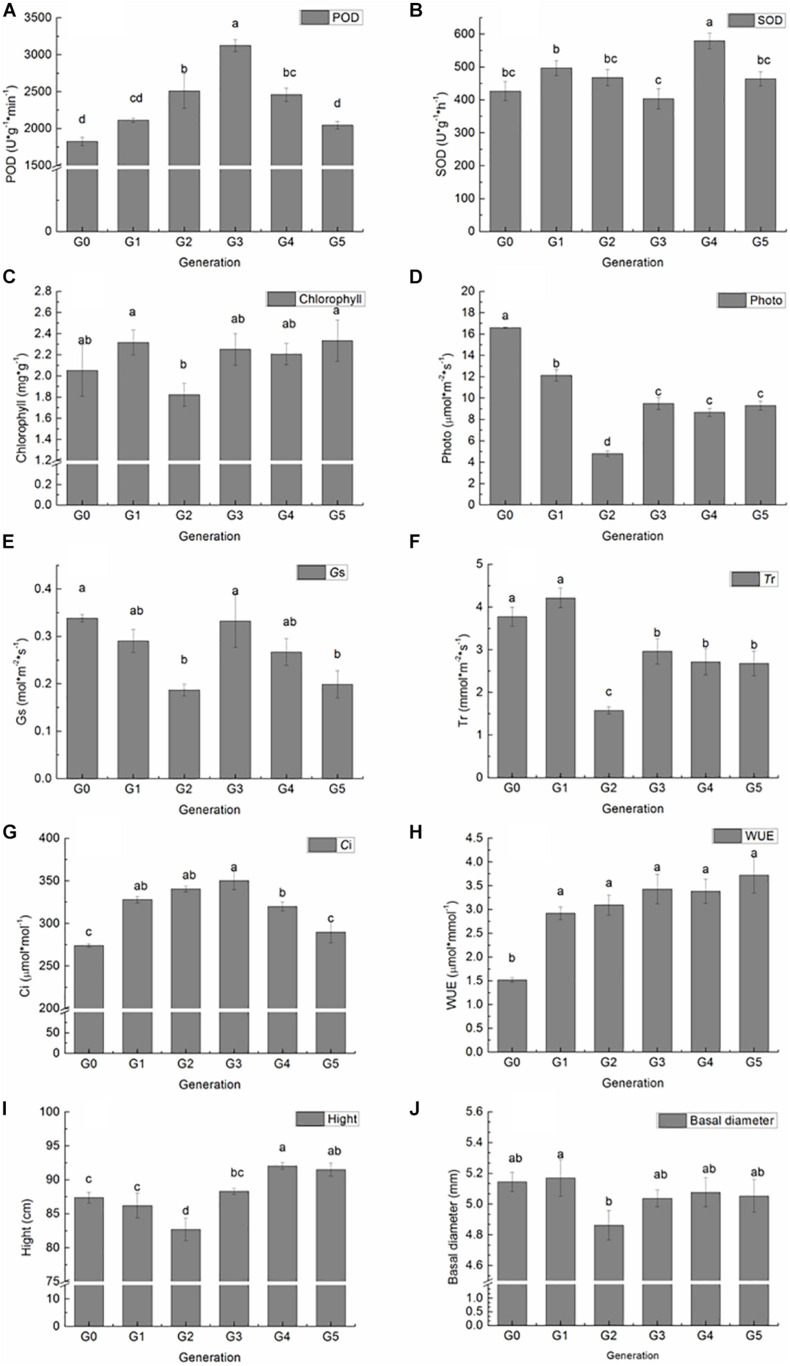
Variation in *P. nigra* physiological and growth parameters among regenerated plants of five generations compared with those of donor plants. **(A)** POD activity. **(B)** SOD activity. **(C)** Chlorophyll content. **(D)** Photosynthetic rate. **(E)** Stomatal conductance. **(F)** Transpiration rate. **(G)** Intercellular CO2 concentration. **(H)** Instant water use efficiency. **(I)** Height. **(J)** Basal diameter. Different letters on columns indicate significant differences at *P* < 0.05.

The POD and SOD activity, total chlorophyll, photosynthetic parameters, height, and basal diameter of G0 plants and all regenerated plants among five generations were measured to investigate the effect of sequential regeneration on plant phenotype (Figure 2). Compared with G0 plants, POD activity increased generationally in G1, G2, and G3 plants, followed by decreases in G4 and G5 plants, finally reaching G0 levels (Figure 2A). A significant difference in POD activity was detected between G2, G3, G4, and G0 plants. However, a similar trend was not observed in SOD activity, with G3 plants having lower and others having higher POD activity than G0 plants; a significant increase in SOD activity was detected only in G4 plants (Figure 2B). Total chlorophyll content increased slightly in G1 plants, declined in G2 plants, increased in G3 plants, and then remained stable in G4 and G5 plants; however, no significant differences were detected between regenerated plants and donor plants (Figure 2C). Pn and Tr values of all regenerated plants were significantly lower than those of donor plants, with the lowest values among G2 plants (Figures 2D,F). Gs decreased significantly in G2 and G5 plants (Figure 2E). Ci increased significantly in G1 plants, continued to rise in G2 plants, peaked in G3 plants, and then decreased in G4 plants; levels became similar to those of donor plants in G5 (Figure 2G). WUE was significantly higher in all regenerated plants than in donor plants (Figure 2H).

Height and basal diameter were measured at the end of the growth season. G4 and G5 plants were significantly taller than donor plants, whereas G2 plants were significantly shorter than donor plants (Figure 2I). Except for G2 plants, which were significantly lower in basal diameter, there were no significant differences among plants of different generations (Figure 2J).

Significant positive correlations (*P* < 0.05) were detected between methylation level and Tr and between methylation level and Pn. A significant negative correlation was found between methylation level and POD activity and between methylation level and WUE (Table 3).

**TABLE 3:** Correlation coefficients between the DNA methylation levels and phenotypic parameters of *P. nigra* N46 regenerated plants of five generations.

	**POD**	**SOD**	**Total chlorophyll**	**Pn**	**Gs**	**Ci**	**Tr**	**WUE**	**Height**	**Basal diameter**
Total methylation level (%)	−0.549*	−0.258	0.810^†^	0.810^†^	0.427	−0.405	0.664^†^	−0.756^†^	−0.292	0.412
Fully methylation level (%)	−0.553*	−0.192	0.766^†^	0.766^†^	0.488*	−0.342	0.685^†^	−0.594^†^	−0.220	0.525*
Hemi-methylation level (%)	−0.498*	−0.288	0.775^†^	0.775^†^	0.340	−0.345	0.589^†^	−0.821^†^	−0.342	0.286

*P < 0.05, ^†^P < 0.01.

## Discussion

Tissue culture conditions, especially the differentiation process can cause DNA methylation changes in plants (Gao et al., 2010; Stroud et al., 2013). Previous studies showed that regenerated plantlets generally had lower methylation levels than donor plants, and demethylation has been reported in regenerated plants of *P. trichocarpa* (Vining et al., 2013), maize (Kaeppler and Phillips, 1993; Stelpflug et al., 2014), cocoa (Rodríguez López et al., 2010), *Malus xiaojinensis* (Huang et al., 2012), rose (Xu et al., 2004), and oil palm (Kubis et al., 2003) etc. These results are consistent with those of the present study; we determined that the regeneration process decreased DNA methylation in *P. nigra* regenerants. We also found evidence that DNA demethylation and methylation had occurred in CCGG sites of *P. nigra* regenerated plants during the regeneration process, and that more frequent demethylation resulted in decreased methylation levels among regenerated plants.

Demethylation in rice regenerated plants has been suggested to be stochastically induced at the tissue culture step (Stroud et al., 2013), such that variation in the methylation rate in plantlets increases with tissue culture time. For example, higher methylation variation rates have been observed in secondary *Clivia miniata* plantlets than in primary plantlets (Wang et al., 2012). Third-callus hop subcultures exhibited higher epigenetic distance from controls than plants from first- and second-callus subcultures (Peredo et al., 2006). In the regenerated plants of our study, the total methylation level of G1 plants was significantly lower than that of donor plants, and that of G2 plants was significantly lower than that of G1 plants; no significant differences were observed among G2–G5 plants. Thus, methylation status generally decreased in first-generation regenerated plants and continued to decrease dramatically as regeneration time increased in poplar. However, methylation levels of plants in later generations (G3–G5) did not always decrease as expected, instead becoming stable after the second regeneration. Therefore, some sites in the plant genome may be more sensitive to the regeneration process, changing their methylation status during the first few generations, and then becoming stable throughout subsequent generations.

Somatic variation has long been observed in field and *in vitro* plant propagation (Meyer, 1980), and more often in plantlets regenerated from calli, which is a process thought to mainly cause DNA methylation changes (Wang et al., 2012; Stroud et al., 2013; Duarte-Ake et al., 2016). *M. xiaojinensis* leaf morphology was observed to vary during tissue culture (Huang et al., 2012); phenotypic variation in oil palm was observed to occur widely and unpredictably following *in vitro* culture (Eeuwens et al., 2002); abnormal flowers, fruits, and ultimately decreased oil yield were observed in regenerated palms (Jaligot et al., 2011). In poplars, variation in growth, leaf phenotype, and gas exchange characteristics of plants regenerated from callus culture have also been reported (Saieed et al., 1994). In this study, significant phenotypic changes were observed in *P. nigra* regenerated plants from certain generations: significantly higher plant height were observed in plants of G4 and G5; significantly lower basal diameter was only observed in G2 plants; significantly greater WUE and significantly lower Pn were observed among all regenerated plants in all five generations; significantly higher POD and SOD activity and Ci, and significantly lower Gs, Tr, and basal diameter were observed in plants within a few generations. Thus, phenotypic variation occurred widely in plantlets sequentially regenerated from poplar leaf explants in this study; however, this variation did not generally increase in magnitude as regeneration time increased. We detected significant correlations between DNA methylation level and some of the examined phenotypes (POD activity, Pn, WUE, and Tr), indicating that this wide and unpredictable phenotypic variation was associated with changes in DNA methylation among poplar regenerated plants.

Apart from the CCGG sites, substantial methylation occurs in plant genomes at the CHG and CHH sites; for example, methylation levels at CG, CHG, and CHH of approximately 24.0, 6.7, and 1.7%, respectively, have been reported for *Arabidopsis* (Cokus et al., 2008); and levels of 41.9, 20.9, and 3.25%, respectively, have been reported for *P. trichocarpa* (Feng et al., 2010). In the present study, we used the MSAP method to detect DNA methylation changes in the genome of plantlets regenerated five times from leaf explants from *P. nigra* “N46.” Limited by this method, we detected only changes in the CCGG motif, which represents a subset of cytosine methylation. Genetic changes (e.g., single-nucleotide polymorphisms or indels) and other epigenetic changes (e.g., histone tail modification or siRNA-based gene silencing) may have happened within the genomes of these poplar regenerated plants too. The sequential regeneration process induced the wide phenotypic variations in poplar regenerated plants and these variations observed may have caused at least partially by the DNA methylation changes.

## Data Availability Statement

The original contributions presented in the study are included in the article/Supplementary Material, further inquiries can be directed to the corresponding author/s.

## Author Contributions

WZ drafted the original manuscript. WZ and YW performed the experiments and analyzed the data. BZ, XS, SD, SZ, SW, and LW were involved in devising and directing the experiments and proofreading the manuscript. BZ contributed to the funding acquisition and edited the final manuscript. All authors reviewed the manuscript and agreed to the publication of this manuscript.

## Conflict of Interest

The authors declare that the research was conducted in the absence of any commercial or financial relationships that could be construed as a potential conflict of interest.

## Supplementary Material

Supplementary Figure 1 Electrophoretogram of MSAP.
