# Supplementary figures and images for: Assessment of Epigenetic and Phenotypic Variation in Populus nigra Regenerated via Sequential Regeneration
Source: Front Plant Sci. 2021 Jul 6;12:632088. doi: 10.3389/fpls.2021.632088 (PMC8290414; doi:10.3389/fpls.2021.632088)

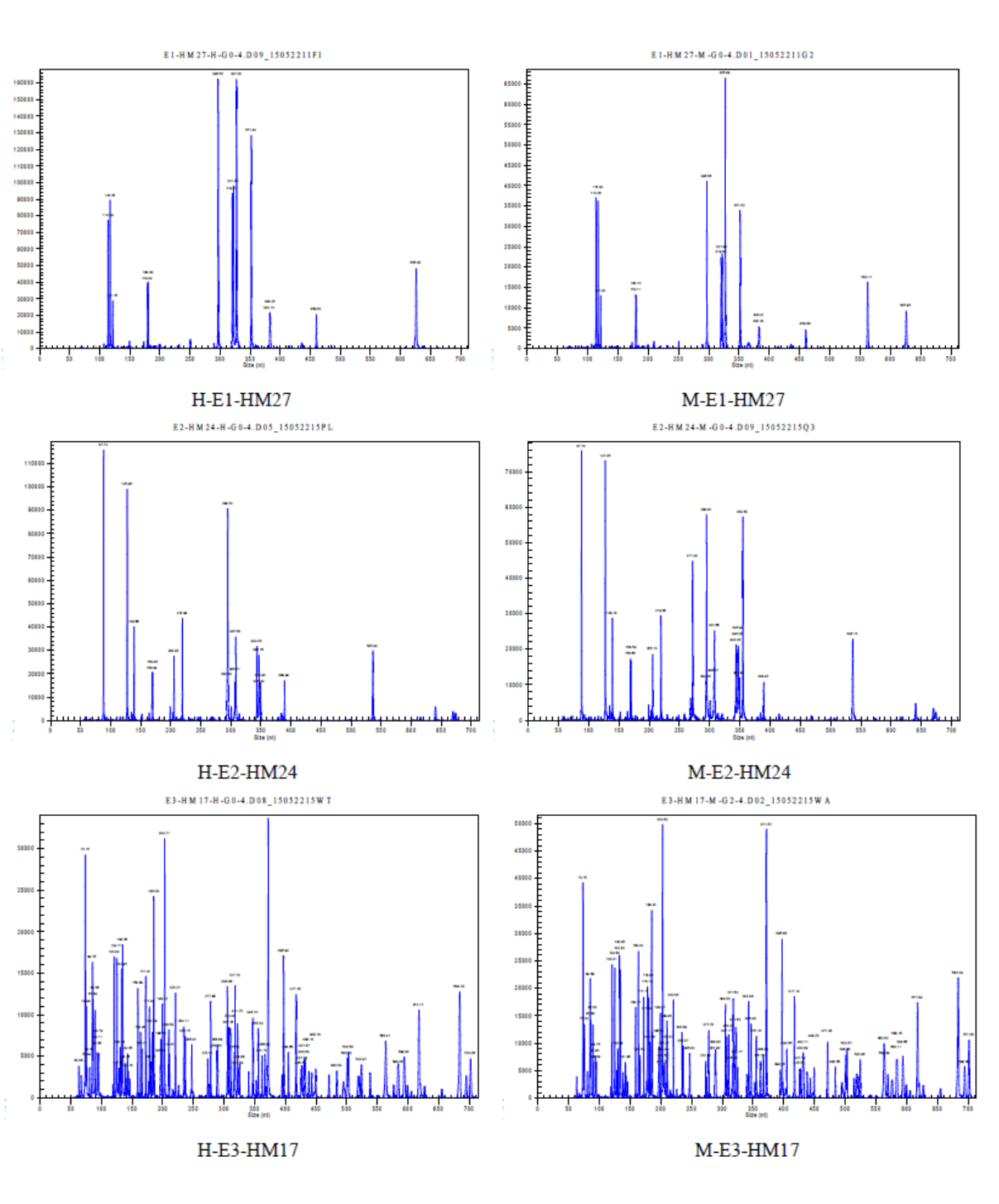

Supplement: Supplementary Figure 1 — Electrophoretogram of MSAP. [file Image_1.tif]
